# Standard peroral endoscopic myotomy combined with open peroral endoscopic myotomy for refractory sigmoid-type achalasia

**DOI:** 10.1055/a-2420-7769

**Published:** 2024-10-14

**Authors:** Fu Guan, Boying Liu, Qunji Zhang, Shengbing Wang

**Affiliations:** 1 608523 Gastroenterology, Meizhou Peopleʼs Hospital, Meizhou, China; 2 608523 Center of Scientific Research and Experiment, Meizhou Peopleʼs Hospital, Meizhou, China

Peroral endoscopic myotomy (POEM) is a feasible, safe, and effective endoscopic treatment for achalasia 1 2 3 . However, standard POEM has limitations when managing sigmoid-type achalasia due to the complexity of anatomical structure in this type of achalasia 4 . We report a case of refractory sigmoid-type achalasia that was successfully treated with a combination of standard POEM and open POEM.

A 66-year-old woman with a 20-year history of recurrent dysphagia was admitted to our hospital. Meglumine diatrizoate esophagram and gastroscopy revealed sigmoid-type achalasia ( Fig. 1 ), and she was subsequently diagnosed with type III achalasia. Standard POEM was initially performed under general anesthesia with endotracheal intubation. A submucosal incision was made 12 cm above the esophagogastric junction (EGJ), and a submucosal tunnel was created through a longitudinal incision of the mucosal and submucosal layers of the posterior wall ( Fig. 2 **a** ). However, the mucosal surface of the tunnel ruptured, and the waterjet nozzle became blocked when the tunnel extended to the S-shaped esophageal bend, located 4 cm from the EGJ, making it impossible to continue ( Fig. 2 **b** ). As a result, we terminated the standard POEM prematurely and proceeded with open POEM ( Video 1 ). This involved a full-thickness incision of the esophageal muscular layer, extending from 7 cm to 3 cm above the EGJ through the tunnel ( Fig. 2 **c, d** ). The mucosal, submucosal, and muscular layers were dissected longitudinally from 3 cm above to 2.5 cm below the EGJ, along the posterior wall of the lower S-shaped esophagus ( Fig. 2 **e, f** ). Finally, titanium clips were used to close the mucosal rupture and the tunnel entrance ( Video 1 ). The patient was discharged 5 days after surgery and followed up for 1 year without recurrence ( Fig. 3 ).

**Fig. 1:**
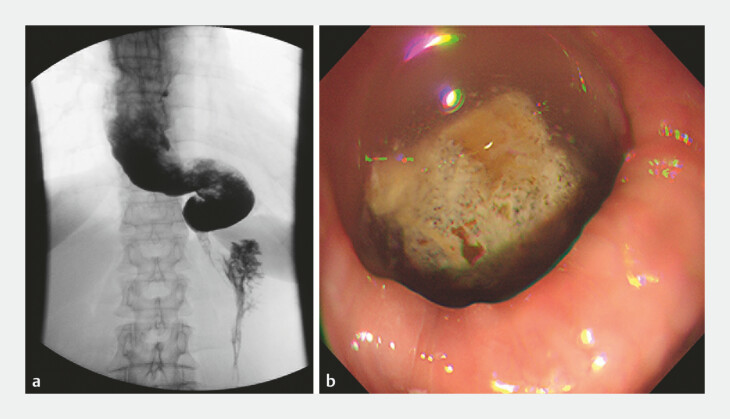
Appearance of sigmoid-type achalasia. **a** Esophagram showing sigmoid-type changes in the lower esophagus. **b** Endoscopic view of food and fluid retention in the esophageal lumen.

**Fig. 2:**
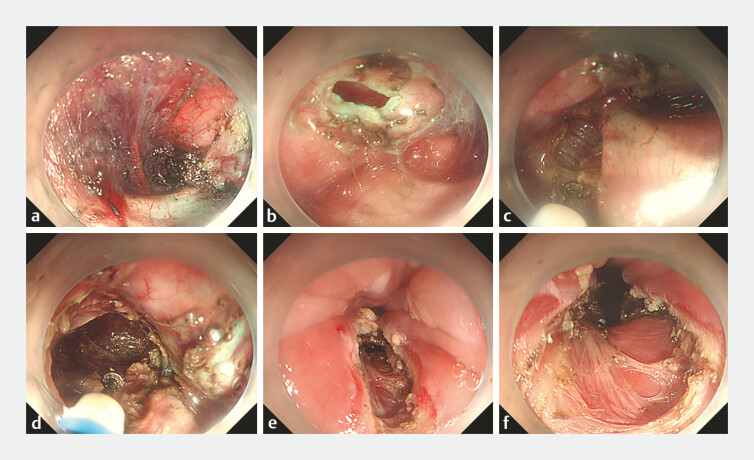
Endoscopic treatment of sigmoid-type achalasia. **a** A submucosal tunnel was established. **b** The mucosal surface of the tunnel ruptured. **c, d** Full-thickness incision of the esophageal muscular layer was made in the tunnel ( **c** ) and extended ( **d** ). **e, f** Esophageal mucosal ( **e** ), submucosal, and muscular layers ( **f** ) were dissected longitudinally in the S-shaped lower esophagus.

**Fig. 3:**
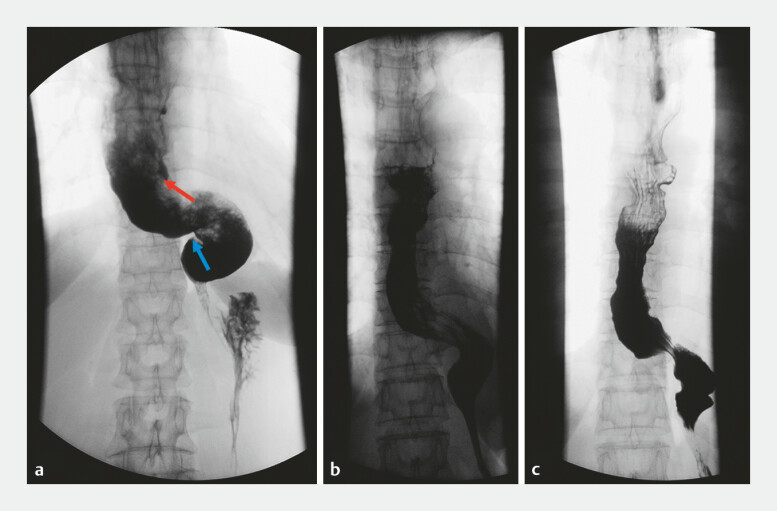
Preoperative and postoperative esophagrams. **a** Preoperative esophagram is shown. Standard peroral endoscopic myotomy (POEM) was performed at one end of the S-shaped esophagus (red arrow) and open POEM was performed at the other end (blue arrow). **b, c** Esophagrams performed at 3 days ( **b** ) and 1 month ( **c** ) postoperatively.

Standard peroral endoscopic myotomy (POEM) combined with open POEM for refractory sigmoid-type achalasia in a 66-year-old woman with a 20-year history of recurrent dysphagia.Video 1

Endoscopy_UCTN_Code_CPL_1AH_2AZ_3AZ

## Notes

Endoscopy E-Videos https://eref.thieme.de/e-videos
*E-Videos* is an open access online section of the journal *Endoscopy* , reporting on interesting cases and new techniques in gastroenterological endoscopy. All papers include a high-quality video and are published with a Creative Commons CC-BY license. Endoscopy E-Videos qualify for HINARI discounts and waivers and eligibility is automatically checked during the submission process. We grant 100% waivers to articles whose corresponding authors are based in Group A countries and 50% waivers to those who are based in Group B countries as classified by Research4Life (see: https://www.research4life.org/access/eligibility/ ). This section has its own submission website at https://mc.manuscriptcentral.com/e-videos .
